# Significance of variation in basal metabolic rate in laboratory mice for translational experiments

**DOI:** 10.1007/s00360-021-01410-9

**Published:** 2021-09-30

**Authors:** Paweł Brzęk, Andrzej Gębczyński, Piotr Selewestruk, Aneta Książek, Julita Sadowska, Marek Konarzewski

**Affiliations:** Faculty of Biology, University of Białystok, Ciołkowskiego 1J, 15-245 Białystok, Poland

**Keywords:** Basal metabolic rate, Thermoneutrality, Mouse, Human, Housing temperature, Spontaneous physical activity

## Abstract

The basal metabolic rate (BMR) accounts for 60–70% of the daily energy expenditure (DEE) in sedentary humans and at least 50% of the DEE in laboratory mice in the thermoneutral zone. Surprisingly, however, the significance of the variation in the BMR is largely overlooked in translational research using such indices as physical activity level (PAL), i.e., the ratio of DEE/BMR. In particular, it is unclear whether emulation of human PAL in mouse models should be carried out within or below the thermoneutral zone. It is also unclear whether physical activity within the thermoneutral zone is limited by the capacity to dissipate heat generated by exercise and obligatory metabolic processes contributing to BMR. We measured PAL and spontaneous physical activity (SPA) in laboratory mice from two lines, divergently selected towards either high or low level of BMR, and acclimated to 30 °C (i.e., the thermoneutral zone), 23 or 4 °C. The mean PAL did not differ between both lines in the mice acclimated to 30 °C but became significantly higher in the low BMR mouse line at the lower ambient temperatures. Acclimation to 30 °C reduced the mean locomotor activity but did not affect the significant difference observed between the selected lines. We conclude that carrying out experiments within the thermoneutral zone can increase the consistency of translational studies aimed at the emulation of human energetics, without affecting the variation in physical activity correlated with BMR.

## Introduction

Recently, many studies have considered what the optimal ambient temperature for experiments on laboratory mice should be to ensure that the results are relevant to humans (Overton 2010; Cannon and Nedergaard 2011; Karp 2012; Speakman and Keijer 2012; Maloney et al. 2014; Ganeshan and Chawla 2017; Fischer et al. 2018; Hankenson et al. 2018; Reitman 2018; Keijer et al. 2019). This is an important translational problem, as ambient temperature can significantly affect the results of experiments on mice (reviewed in Maloney et al. 2014; Ganeshan and Chawla 2017; Hylander et al. 2017; Morton et al. 2019). The high body-surface to mass ratio leads to an increase in heat loss in mice (Gordon 2012; Reitman 2018). Therefore, typical temperatures maintained in animal facilities (ca. 23 °C) effectively represent cold stress for mice, whereas, humans typically live within or just below the thermoneutral zone (TNZ; Karp 2012; Ganeshan and Chawla 2017). Several recent studies argued that to reliably emulate the energy budget of humans, single-housed laboratory mice should be maintained at 23–25 °C (i.e., near the typical temperature in animal facilities; Speakman and Keijer 2012), 28–30 °C (i.e., TNZ of mice; Fischer et al. 2018), or some intermediate temperature (25.5–27.6 °C; Keijer et al. 2019).

In the controversy outlined above, we note a surprisingly overlooked problem: the effect of variation in basal metabolic rate (BMR) and its mechanistic associations with other traits directly linked to energy budgets. In sedentary humans, the BMR exceeds 60% of the total energy expenditures (Fig. 2a in Garland et al. 2011). It is, therefore, unsurprising that the link between the variation in BMR and such factors as weight gain and the development of metabolic syndrome has received much attention (Ravussin et al. 1988; Astrup et al. 1996; Weinsier et al. 2000; Buscemi et al. 2005, 2007; Vogels et al. 2005; Lazzer et al. 2010; Hohenadel et al. 2019). Similarly, the BMR is the most important component of the energy budget in mice maintained in the TNZ (Abreu-Vieira et al. 2015) and varies significantly between different strains of mice (Konarzewski and Diamond 1995). Because BMR quantifies obligatory heat production, it should directly affect how the animals respond to ambient temperature, particularly within or just below TNZ.

Two traits that are particularly likely to covary with BMR are the daily energy expenditure (DEE) and spontaneous physical activity (SPA). One of the key problems in the above-outlined controversy over the optimal ambient temperature in translational experiments in mice is its effect on the ratio of DEE to BMR (referred to as the physical activity level, PAL). Most authors agree that laboratory mice should be exposed to an ambient temperatures ensuring PAL at the level of 1.7–1.8, which is equivalent to that found in humans (Speakman and Keijer 2012; Fischer et al. 2018, 2019; Keijer et al. 2019). However, if variation in BMR correlates with other components of the energy budget, it is also likely to modulate the PAL. For example, if a high BMR correlates with reduced dedicated thermogenesis because of a higher obligatory heat production, then the animals having a high BMR do not need to elevate their metabolic expenditure over BMR as much as individuals with a lower BMR. This should result in a lower PAL in high BMR individuals.

An even more important role can be played by the SPA, which represents an important component of energy expenditure in laboratory animals, particularly in the TNZ (Gordon 1993; Garland et al. 2011). A human analog of SPA is known as NEAT (nonexercise activity thermogenesis), which accounts for a significant part of the energy expenditure in everyday activity and is therefore crucial in the regulation of body mass and the search for an effective treatment for obesity or metabolic syndrome (Levine 2007; Teske et al. 2012; Huffman et al. 2014; Kotz et al. 2017). We are not aware of studies investigating the links between genetically determined BMR and SPA in humans. However, there is a general positive link between BMR/RMR and SPA in animals (reviewed in Careau and Garland 2012; Biro et al 2018), which is likely to be mediated by an ambient temperature. For example, heat generated by behavior can be substituted for thermogenesis at low ambient temperatures (Humphries and Careau 2011), and thus the relative costs of an elevated SPA can be reduced. On the other hand, a positive correlation between BMR and SPA can be weakened under high ambient temperatures because of insufficient dissipation of heat generated simultaneously by both high BMR and an intense SPA (Speakman and Keijer 2012). Therefore, the weakening of a positive correlation between BMR and SPA with an increasing ambient temperature may be an important factor affecting the translational relevance of experiments on mice.

To evaluate the significance of variation of BMR for translational research, we studied the effect of acclimation to different ambient temperatures on DEE and SPA in two lines of laboratory mice, selected divergently towards either a high (H-BMR) or a low (L-BMR) level of BMR. The difference between these two lines in body-mass-specific BMR exceeds 50% (see “Animals and experimental groups” section), and thus, their BMRs are likely to encompass the entire variation for this characteristic manifested by mouse strains used in translational research. Furthermore, although these two lines differ significantly in the magnitude of obligatory (i.e., BMR-based) heat production, they have the same thermal conductance (Sadowska et al. 2019). As a result, L-BMR mice must rely relatively more on the sources of heat other than BMR (such as NST) at temperatures below 30 °C (Brzęk et al. 2019). In addition, the H-BMR mice show a higher SPA than L-BMR mice at 23 °C, and the magnitude of this difference suggests that SPA is genetically correlated with BMR (Brzęk et al. 2016). Thus, both lines are an excellent model for investigating the effect of variation in BMR on traits directly relevant to translational studies on energy expenditures.

To gain further insight into the significance of variation in BMR on choosing the appropriate ambient temperature for translational research, we compared DEE, PAL and SPA measured in both lines at 30 °C (i.e., within the TNZ) and at 23 °C (the typical maintenance temperature in mouse facilities). Furthermore, we also quantified these parameters at 4 °C to evaluate the significance of the nonlinearity of the effect of temperature below the TNZ on those traits.

## Materials and methods

### Animals and experimental groups

A detailed description of the procedures during the selection experiment is given in Książek et al. (2004). The present study was carried out on males from generation F49 of the selection experiments. In this generation the BMR (body-mass corrected LS mean ± SE) was 40.8 ± 0.91 ml O_2_ h^−1^ in the L-BMR line and 67.5 ± 0.87 ml O_2_ h^−1^ in the H-BMR line. Mice (5–6 months old at the beginning of the experiment) were assigned randomly to three different temperature regimes: maintained at 23 °C (i.e., the normal rearing temperature used during the course of experimental selection), acclimated to 30 °C, and acclimated to 4 °C. We note that while 30 °C is within the TNZ for both lines (A. Gębczyński, pers. obs.), 23 °C presumably represents a bigger cold stress for the L-BMR line than the H-BMR line (because of the lower BMR), and 4 °C is likely to represent a severe cold stress for both lines.

We used 16–20 mice from each selected line for each treatment. All mice were kept individually in cages supplied with standard bedding (woodchips), 12L:12D and unlimited access to water and food (standard laboratory chow; energy content: 16.1 kJ/g; Labofeed H, Kcynia, Poland). Experimental treatments lasted 1 month (range of 29–33 days). The mice assigned to the different temperature treatments did not differ in body mass (effect of temperature treatment and all interactions between the effects of temperature treatment, time course of the experiment, and line affiliation were non-significant).

### Measurements of metabolic rate

During the last 24 h of experimental treatment, energy expenditure and SPA were quantified. Energy expenditure was measured by means of open-flow respirometry. The whole cage with the animal was placed into a tight 30 × 22 × 21 cm container made of transparent polycarbonate (Macrolon) that closely fit the cage’s size, with a steady air flow (400 ml per minute). We simultaneously used three respiratory systems: one system measured three individuals in three separate channels, with automatic switching between channels and short background measurements between every channel. This system resulted in 11 min of continuous measurements of every individual during each hour. We excluded from our dataset data collected between 08:00 and 10:00 am because of disruptive human presence in the chamber that may affect animal activity and energy expenditure (see below for further explanation).

**Fig. 1:**
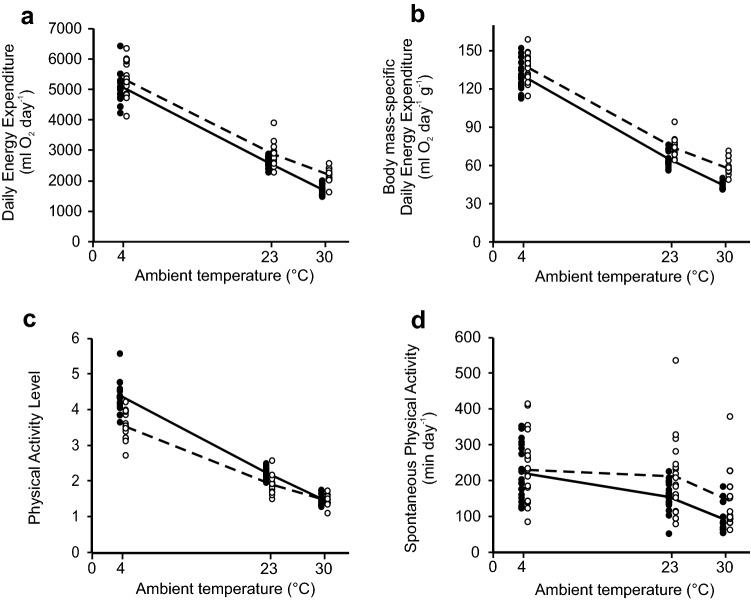
Original data points for DEE **a**, body mass-specific DEE **b**, PAL **c**, and SPA **d** in mice from both selected lines, acclimated to ambient temperatures of 4, 23, and 30 °C. Each point indicates one individual mouse, and the lines connect mean values. L-BMR line—black dots and solid line; H-BMR line—white dots and dashed line. Values depicted on panel 1b (i.e. DEE divided by body mass) are presented only for illustration purpose, as DEE was controlled for body mass in statistical analysis by means of ANCOVA

We quantified the following estimates of energy expenditure:

1. for each mouse in group acclimated to 30 °C, we chose one single continuous 11 min measurement with the lowest mean oxygen consumption, which was closest to the BMR. We refer to this measurement as to RMR because not all requirements of BMR measurements were fulfilled (e.g. mice were not fasted). We measured the metabolic rate of each individual mouse for only 20% of each hour (see above). However, our RMR estimates clustered in the afternoon, as can be expected in mice reared at the TNZ (compare with Fischer et al. 2018), and were similar to BMR measured in selected lines in recent generations of selection experiment (P. Brzęk, pers. observ.). Thus, they represented a reliable proxy of BMR of studied mice;
2. DEE, defined as the total daily oxygen consumption. To calculate DEE, we extrapolated oxygen consumption measured during 11 min period to the whole hour, and subsequently summed those values over all hours. Because we excluded two hours from the day (see above), we substituted those missing values for each individual mouse with mean values calculated for the remaining daylight hours (we checked that our final conclusions were not affected by this procedure);
3. PAL, defined as the DEE/BMR ratio (BMR was multiplied by 24 to ensure that both variables quantify energy expenditure during the same time period). We did not quantify the BMR at 30 °C in mice acclimated to 23 and 4 °C because this would disrupt acclimation to those temperatures; however, since the groups acclimated to different ambient temperatures did not differ in mean body mass, we used the mean RMR measured in mice acclimated to 30 °C as a proxy of BMR for all temperatures. We note that RMR measured at 30 °C was the same in mice acclimated to 30 and 21 °C (Fischer et al. 2018); thus, our assumption should be valid at least for mice acclimated to 30 and 23 °C. Therefore, we divided the DEE calculated separately for each individual mouse at each temperature by the average RMR calculated for the particular line in the group acclimated to 30 °C. The mean values of PAL found in our experiment (Fig. 1c) matched well with the results obtained by Keijer et al. (2019) (PAL = 1.66 at 30 °C and PAL = 2.13 at 21.3 °C) for a similar, 10-min long estimate of BMR.

### Quantification of SPA

The total SPA was quantified by means of passive infrared sensors as described in Brzęk et al. (2016). This assay was not carried out in six mice acclimated to 23 °C (3 from each line) because of sensor malfunction. All disruptive activities related to the human presence in a chamber occurred between 08:00 and 10:00 am; these data were excluded from our dataset and substituted in the same way as described above for DEE (again, this treatment did not affect our final conclusions). We measured all daily activities in sedentary mice, and thus our SPA corresponds to what is usually defined in humans as non-exercise activity thermogenesis (NEAT), whereas, SPA in humans usually has a narrower definition (Dullo et al. 2012). However, because definitions of SPA/NEAT are rather vague (Garland et al. 2011), we will subsequently use the abbreviation SPA for all non-exercise activities in both mice and humans for the sake of brevity.

### Statistics

We analyzed RMR in mice acclimated to 30 °C by means of ANCOVA, with the effect of line affiliation as the main factor and the effect of body mass as a covariate. We compared DEE, PAL, and SPA in mice acclimated to all three ambient temperatures by means of one-way ANCOVA, with the effect of line affiliation as the main factor, the effect of ambient temperature (both linear and square terms to check for non-linearity) coded as covariates and tested along with their interactions with the line affiliation, measurement channel as a random variable (with nine levels, representing separate respiratory system channels or infrared sensors), and (for DEE) body mass as a covariate. All factors other than the effects of line affiliation and ambient temperature were included in the final models only when significant. When a significant interaction between the effects of line affiliation and ambient temperature was found, we compared the selected lines separately for each ambient temperature by means of simple ANOVA or (for DEE) ANCOVA with body mass as a covariate. To control for multiple comparisons, we applied the Bonferroni correction, dividing the conventional *p* = 0.05 by 3 (i.e. the number of separate comparisons between selected lines for ambient temperatures). The homogeneity of variance was tested by means of Bartlett’s test and variables were log-transformed when necessary. All analyses were carried out using SAS 9.4 software (SAS Institute, Cary, NC, USA).

## Results

Mice selected for low BMR and acclimated to 30 °C had lower RMR than mice from the line selected for high BMR (ANCOVA; F 1, 29 = 81, *p* < 0.0001, body mass was significant as covariate; least-square means ± SE: L-BMR: 46.8 ± 1.30 ml O_2_ h^−1^, H-BMR: 63.1 ± 1.30 ml O_2_ h^−1^). We found a significant interaction between the effects of ambient temperature and line affiliation on DEE (Table 1). Thus, genetically determined between-line differences in BMR resulted in an uneven, line-specific increase in DEE with decreasing ambient temperature. To gain further insight into those line-specific response, we compared both lines separately within each temperature treatment by means of ANCOVA with body mass as a covariate. DEE was consistently higher in the H-BMR mice at each ambient temperature (*p* ≤ 0.0001, Fig. 1a, b), though the significant interaction term suggests that the magnitude of this difference was relatively larger at 30 °C (LSM ± SE means for L-BMR and H-BMR lines, respectively: 30 °C: 1682 ± 80.8 ml O_2_ day^−1^ and 2265 ± 80.9 ml O_2_ day^−1^, 23 °C: 2506 ± 79.7 ml O_2_ day^−1^ and 2901 ± 80.6 ml O_2_ day^−1^, 4 °C: 5054 ± 72.0 ml O_2_ day^−1^ and 5346 ± 74.1 ml O_2_ day^−1^).

**Table 1:** Summary of ANCOVA for mice acclimated to 4 °C, 23 °C and 30 °C, with the effect of ambient temperature as the continuous covariate (ambient temperature^2^ indicates the square term)

	Line affiliation		Ambient temperature		Ambient temperature^2^		Interaction line affiliation × ambient temperature		Interaction line affiliation × ambient temperature^2^	
	*F*	*p*	*F*	*p*	*F*	*p*	*F*	*p*	*F*	*p*
DEE	4.84	0.03	25.5	<0.0001	11.4	0.001	2.44	0.12	5.92	0.017
PAL	7.41	0.008	11.8	0.0009	11.2	0.0011	2.85	0.09	5.28	0.024
SPA	13.7	0.0004	5.23	0.024	13.4	0.0004	ns	ns	ns	ns

Degrees of freedom (*df*) for all factors were 1, 97 for DEE, and 1, 98 for PAL and SPA. Body mass was significant as the covariate for DEE (*F* = 52, *p* < 0.0001). Measurement channel was significant as the random variable for SPA (*p* = 0.045)

Similarly, we found significant interactions between the effects of line affiliation and ambient temperature for PAL (Table 1; Fig. 1c). ANOVAs carried out separately for each ambient temperature revealed that PAL did not differ between the lines at 30 °C (*p* = 0.55), but was significantly higher in the L-BMR mice at 23 and 4 °C (*p* < 0.0001 at both temperatures).

The SPA of both mouse lines increased more conspicuously between 30 and 23 °C than between 23 and 4 °C (Fig. 1d). This nonlinearity resulted in a high statistical significance of the squared value of an ambient temperature term used as a covariate in the ANCOVA analysis (Table 1). However, the lack of significant interaction between effects of line affiliation and ambient temperature revealed that an increase in SPA was similar in scope in both lines (Table 1), with the SPA significantly higher in the H-BMR mice at all temperatures (Fig. 1d).

## Discussion

We demonstrated that the systematic variation in BMR and related traits is a fundamental but overlooked factor that should be considered when choosing an ambient temperature for translational experiments in laboratory mice.

Here, we found that the average PAL did not differ between the two lines of mice, selected for either high or low BMR, when all measurements were carried out at 30 °C but became higher in the L-BMR line at the lower ambient temperatures (Fig. 1c). We also found significant between-line differences in the DEE across the whole temperature gradient (Fig. 1a, b), that arose as a correlated response to selection on the BMR, by definition carried out only within the TNZ. However, a significant interaction between the effects of line affiliation and ambient temperature on DEE indicates that at ambient temperatures below 30 °C, mice from the H-BMR line did not need to elevate their metabolic rate as much as the mice from the L-BMR line that had to rely comparatively more on heat generated by SPA or dedicated thermogenesis. As a result, PAL of the L-BMR mice became higher.

It is generally agreed that the ambient temperature for translational experiments in mice should emulate the typical PAL observed in humans, i.e., 1.7–1.8 (Speakman and Keijer 2012; Fischer et al. 2018, 2019; Keijer et al. 2019), and the only issue discussed typically is whether this PAL is reached within or below the TNZ (which reflects mainly the methodology of the BMR measurements; Keijer et al. 2019). Therefore, even though we did not estimate the exact lower limit of the TNZ in the studied lines, our results could be seen as clear evidence that the optimal ambient temperature for translational experiments should be lower than the TNZ but higher than 23 °C (Fig. 1c; similar to the recommendation by Keijer et al. 2019). However, our results demonstrate that the discussion on the impact of ambient temperature on PAL has overlooked the significance of variation in BMR that, as we have shown, can affect PAL in an ambient-temperature-specific manner. We assert that this effect may have such profound consequences that translational experiments on mice should be carried out within the TNZ (in practice, at 30 °C). Below we justify our rationale at length.

**Fig. 2:**
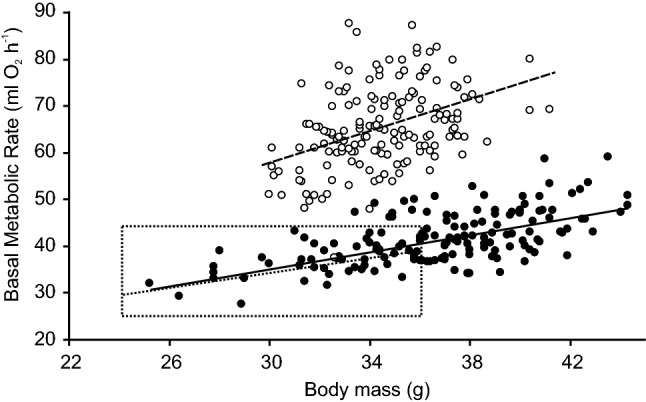
BMR plotted against body mass in males from generation F49 (used in the present experiment). Each point indicates one individual mouse, and the lines indicate regression lines. L-BMR line—black dots and solid line; H-BMR line—white dots and dashed line. The dotted square indicates the range of variation in body mass and BMR for 57 males of the C57BL/6 strain depicted in Fig. 4a in Mitchell et al. (2017), and the dotted line indicates the line of regression for those data

We are not aware of any other study of the effect of the differences in BMR on PAL across three different ambient temperatures (we only note that Fig. 2b–d in Keijer et al. (2019) suggests that inter-individual variation in PAL in C57BL/6 mice is typically narrower at 30 °C than at lower ambient temperatures). However, we hypothesize that Fig. 1c depicts a general pattern that should be representative to any strains of mice with different BMR, as well as to the within-strain variation in BMR. Our selection experiment changed the values of BMR in both directions when compared to the non-selected Swiss mice (Gębczyński and Konarzewski 2009; Maciak et al. 2014). Thus, the relative difference in mean BMR between our selected lines (65% for males, 57% for females in generation F49) likely encompassed the range of the variation in BMR that could be expected between the mouse strains used in biomedical research (at least 35%, Konarzewski and Diamond 1995; see also Fig. 2). At the same time, the within-strain variation in BMR in C57BL/6 mice with the same body mass can surpass 70% (females: Fig. 1 in Johnston et al. (2007); males: Fig. 4a in Mitchell et al. (2017)). Nevertheless, we have shown that two lines of mice with a very large (65%) difference in BMR can have the same PAL at 30 °C (Fig. 1c). Thus, we suggest that carrying out biomedical experiments at 30 °C should reduce the variation in PAL between studies and thus improve the reproducibility of their results (Hankenson et al. 2018).

One could argue that the same goal could be achieved by strain-specific adjustment of the ambient temperature to guarantee a PAL = 1.7–1.8. However, accurate and unequivocal estimation of TNZ can be challenging methodologically and the lower critical temperature can be affected by factors like sex or health, yielding even within-strain differences (Gordon 1993, 2012). On the other hand, it is justified to assume that 30 °C lies within TNZ for most of mouse strains (e.g. Table 2 in Speakman and Keijer 2012). We also note that 30 °C is already frequently used in translational studies and thus further use of the same ambient temperature should make results of different experiments more comparable. Thus, for both methodological and logistical reasons the strain-specific adjustment of ambient temperature may be laborious but not significantly better than the use of constant value of 30 °C (unless 30 °C is clearly below TNZ for particular strain). To further strengthen our argumentation let us also point out that the effect of within-strain variation in BMR on PAL below the TNZ is likely to be at least comparable to the effect of the ambient temperature, making such strain-specific adjustment even more difficult. If two mice had the same DEE, then just a 6% difference in BMR could change the value of PAL from 1.7 to 1.8. According to Fig. 2b in Keijer et al. (2019), this is the predicted effect of a 2 °C change in the ambient temperature on the mean PAL, i.e., similar to the difference between the ambient temperatures recommended in some recent papers (e.g., Fischer et al. 2018; Keijer et al. 2019). We predict that if high BMR reduces lower critical temperature, then its effect on PAL should be different for individuals with low or high BMR: those with the high BMR still remain within their TNZ, but those with a low BMR are already below their TNZ and must increase their metabolic rate (strikingly, this pattern also seems to be depicted in Fig. 2b in Keijer et al. (2019): the lowest values of PAL did not differ between the mice measured at 30 and 27 °C, but the upper border of variation in PAL was already elevated at 27 °C). Clearly, 30 °C is more likely to fall within the TNZ of most individuals in most mouse strains used in translational studies, and thus reduce both inter- and intra-strain variation in PAL.

Finally, the energy budgets of mice and humans below thermoneutrality are very different, and the effect of variation in BMR on thermoregulatory needs can further reinforce this difference. In mice, thermoregulation plays a more important role than in humans (Reitman 2018), and PAL in small mammals is typically larger than in humans, which has been interpreted as the effect of the considerable cost of thermoregulation, relatively higher than the cost of the physical activity in our species (Westerterp and Speakman 2008). Thus, below TNZ, the same absolute values of PAL in humans and mice do not guarantee the functional similarity of their energy budgets. SPA-generated heat in laboratory mice is easily substituted for thermogenesis, and any unambiguous apportionment between the costs of SPA and thermoregulation is almost impossible (Virtue et al. 2012). This is best presented by an almost humorous fact: both Fig. 2 in Garland et al. (2011) and Fig. 2b in Abreu-Vieira et al. (2015) show virtually the same (56% of DEE) combined cost of SPA and thermoregulation in mice at 21 °C. However, whereas, the relative percentage of SPA and thermoregulation was estimated to be 39% and 17%, respectively, as reported in Garland et al. (2011), the same values reported in Abreu-Vieira et al. (2015) were 13% and 43%! Complex interactions between locomotor activity and thermoregulation were the reason for the recommendation of thermoneutrality as the preferred ambient temperature for experiments on laboratory mice in another recent study (Blais et al. 2018). The results of our experiment suggest that the variation in BMR can make these interactions even more complicated and thus can only enhance that recommendation.

One of the arguments raised against carrying out experiments on laboratory mice in the TNZ is that the dissipation of SPA-generated heat could pose a significant problem at a high ambient temperature (Speakman and Keijer 2012; Keijer et al. 2019). Because the between-line difference in DEE in our experiment was larger at 30 °C than at 23 °C (Fig. 1a, b), and because both lines did not differ with respect to their thermal conductance (Sadowska et al. 2019), one could expect that the SPA should be particularly likely to be suppressed at 30 °C at a relatively higher rate in the H-BMR line. However, even though the ambient temperature in our experiment indeed exerted a strong effect on the SPA (particularly between 23 and 30 °C; Fig. 1d), the difference between the lines was the same at both of these temperatures. There are at least two plausible mechanisms that could be responsible for this pattern. First, our selection affected the intensity of the SPA rather than the duration of the activity phase (Brzęk et al. 2016), whereas, SPA duration was shown to be the better predictor of food intake in mice than the intensity of SPA (Hiramatsu and Garland 2018). Thus, the H-BMR and L-BMR lines can actually differ little in the energetic cost of SPA and, consequently, the amount of SPA-generated heat. Second, the insulation in mice can depend relatively more on physiological mechanisms (such as changes in tail blood flow) rather than on fur properties (Abreu-Vieira et al. 2015; Fischer et al. 2018), which should enable an easy and flexible heat dissipation, sufficient at least for the level of SPA observed in sedentary mice. Here, we cannot exclude that the H-BMR mice are more effective in using these physiological mechanisms than the L-BMR line. Irrespective of the responsible mechanisms, our results clearly show that even though SPA was indeed reduced by high ambient temperature, any potential heat stress did not preclude the H-BMR mice from expressing their genetically based higher SPA (this conclusion agrees with the results of earlier experiments on other costly traits in the same lines of mice (Książek and Konarzewski 2016; Sadowska et al. 2019). Furthermore, our results suggest that the between-strain differences in SPA are not affected by thermal preferences of mice that can vary during the day (Keijer et al. 2019). We conclude that if a > 50% difference in the mean BMR between the lines does not obviously limit the relative performance of mice at 30 °C, it is also unlikely that the natural variation in BMR or in covarying traits observed in other strains of mice would be hampered by that temperature.

The effect of ambient temperature on SPA in our experiment was non-proportional, as revealed by a significant square term for ambient temperature in the ANCOVA analysis (Table 1). Actually, the SPA of mice acclimated to 23 °C was similar to that in individuals acclimated to 4 °C (i.e., severe cold stress) rather than to 30 °C (Fig. 1d). Thus, the relative effect of ambient temperature below the TNZ on energy expenditure and SPA in mice seems to be very different from that in humans. For example, in humans, a 5–6 °C decrease in ambient temperature within the range of 16–24 °C results in a small (4–6%) though significant increase in DEE and no change in SPA (Westerterp-Plantenga et al. 2002; Celi et al. 2010). In our experiment, a 7 °C difference below the TNZ increased DEE 52% in the L-BMR line and 27% in the H-BMR line (Fig. 1a, b), whereas, SPA was increased 43% in the H-BMR line and 67% in the L-BMR line (Fig. 1d). This is another reason why the mice maintained below the TNZ can be a poor model of human energetics.

Variation in BMR is a natural phenomenon that occurs in both humans and mice. However, we conclude that the effect of variation in BMR on PAL and the energy budget in mice is so different within and below the TNZ that thermoneutrality (what can be safely assumed to represent 30 °C) can be the best choice for experiments on laboratory mice, ensuring a clear interpretation of the results and improving their reproducibility. BMR has the strongest effect on DEE under conditions of thermoneutrality (thus, it is particularly recommended when one examines the effect of BMR on an analyzed trait). Interestingly, in humans, the link between physical activity and DEE is significant for low intensity activities but disappears when DEE is high (Pontzer et al. 2016), though not because of a substitution for thermoregulation but rather due to changes in other components of the energy budget (Pontzer 2015). Thus, the SPA of laboratory mice can be a valid model of at least relatively sedentary (i.e., Western) human populations but only in the TNZ where there is clear link between SPA and DEE. We conclude also that the need for dissipation of heat generated during normal physical activity is not an important argument against maintaining laboratory mice at the TNZ. At worst, a high ambient temperature can indeed affect the mean SPA, but we can still assume that the link between variation in SPA and its correlates (or causes) should be revealed even at 30 °C. On the other hand, when the experiment must be carried out below the TNZ, the variation in BMR can exert so large an effect on PAL that there is no reason to claim that any particular ambient temperature guarantees a better mimicking of the human energy budget than others. Finally, we would like to point out that our conclusions should not be interpreted as a recommendation for a continuous maintenance of laboratory mice within their TNZs. This could create unnecessary logistic burden; however, acclimation of animals to 30 °C prior to planned experiments is a more feasible strategy.

## Data Availability

All data are available upon request.

## Abbreviations

DEE: Daily energy expenditure
BMR: Basal metabolic rate
RMR: Resting metabolic rate
PAL: Physical activity level
SPA: Spontaneous physical activity
L-BMR: Line of mice selected for low basal metabolic rate
H-BMR: Line of mice selected for high basal metabolic rate
TNZ: Thermoneutral zone
